# Under-five mortality among displaced populations in Meheba refugee camp, Zambia, 2008–2014

**DOI:** 10.1186/s13690-016-0161-9

**Published:** 2016-11-21

**Authors:** Nachela Malisenti Chelwa, Rosemary Ndonyo Likwa, Jeremiah Banda

**Affiliations:** Department of Public Health, School of Medicine, The University of Zambia, Lusaka, Zambia

**Keywords:** Underfive mortality, Displaced populations, Refugee camps, Post emmergency phase

## Abstract

**Background:**

Under-five mortality, which is the probability of a child dying before their fifth birthday, is of concern in Zambia as infant and child mortality rates are important social indicators. Displaced population in camps provide a basis for under-five mortality surveillance because detailed registration databases have been developed. Additionally, health data routinely collected on mortality allowed for a review of mortality trends and identification of correlating factors to under-five mortality. Literature suggests a number of factors that influence child mortality including biological, socio-econimic and environmental factors. However, while progress in reducing mortality is evident disparities in under-five mortality trends have been observed.

**Methods:**

The study examined differential levels and trends of under-five mortality with correlating factors in Meheba refugee camp in Zambia which is presently in its post emergency phase. The retrospective cross-sectional study reviewed the ProGres and Health Information System (HIS) databases under-five mortality data for a seven (7) year period (2008–2014) and included all children aged less than five years in each year of review. STATA 12 (including Ordinary Least Squares Regression) and Microsoft Excel 2010 where used for data analysis and computation of findings.

**Results:**

Malaria and respiratory infections accounted for 81 % of under-five deaths while cases of Diarrhoea were responsible for 10 % of reported mortalities. Seventy five percent (75 %) of all mortalities were reported in children aged less than 1 year (<1 year). While no significant variations in mortality were noted as a result of time, increased frequency of visits to health centre significantly (*P* < 0.05) reduced mortalities in children by 3/1000 in each year.

**Conclusion:**

In addition to improving health infrastructure and reducing distances to health facilities, the study also recommends sensitization programmes targeted at ensuring accessibility to health care services for children under-5 years. The study found that increased health centre visitations were associated with reduction in under-five mortality and encourages initiatives targeted at sensitizing communities to seek health care. Furthermore, collaboration between the health systems, community and Non Governmental Organisations (NGOs) is key in addressing higher infant mortality observed. It is envisaged that this will contribute to the reduction in mortality cases and will compliment already existing strategies.

## Background

Under-five mortality is of concern in Zambia as infant and child mortality rates are important social indicators associated with wellbeing or quality of life. Demographic health survey (DHS) data collected between 1992 and 2013 suggests that Zambia has made some progress towards attainment of Millennium Development Goals (MDGs) evidenced, in part, by the reduction of child mortality. Trends in Child mortality have reduced from 191/1000 in 1992 to 75/1000 in 2014. A United Nations International Children’s Emergency Fund (UNICEF) report (2013) highlights that if current trends of under-five mortality continue; the world will not meet Millennium Development Goal 4 – to cut the rate of under-five mortality by two-thirds by 2015.

However, displaced populations present a somewhat different scenario once the post emergency phase is reached. Studies conducted among displaced populations globally suggest that following transition to host country, refugee under five mortality rate (U5MR) reaches rates that are much lower than those of the host nation and community [8]. Mortality data collected consistently and routinely provides information relevant for decision making regarding the health of the population. Refugees are usually at the highest risk of mortality during the period immediately after their arrival in the country [9].

A study of Mozambican refugees hosted in Zimbabwe by the Centre for Disease Control and Prevention (CDC) in 1993 revealed that during July and August 1992, the Crude Mortality Rate (CMR) among the Mozambican refugees who had been in a refugee camp for less than 1 month was 8 per 10,000 populations. This is four times the death rate of refugees who had been in the camp between 1 and 3 months, and 16 times the death rate that is normally reported for non-displaced populations in Mozambique [3].

Another study by Elias et.al suggested that the analysis of mortality trends can be used to evaluate the effectiveness of assistance programs in these camps as infectious disease control in long term camps has proven to be more effective than short term camp structures. The Vietnamese invasion of Cambodia in 1979 saw an influx of refugees into camps in Thailand who arrived physically, mentally and nutritionally exhausted after close to a decade of war [5]. Ten (10) years following the war close to 350,000 refugees with an age distribution skewed towards the very young (23 % under age 5) were still resident in these camps. A study conducted at two of the refugee camps revealed that in the long term camps the estimated infant and U5MR were comparable to those in countries such as Panama and Korea in which there had been no conflict and were considerably lower than the host country Thailand [5].

Studies undertaken on disparities on U5MR in Zambia reveal disparities exist between urban and rural areas and among rural areas themselves owing to the limited access to health care services, poor state of infrastructure and malnutrition, all of which plague refugee camps at initiation. The 2013–2014 ZDHS report findings state that 1 in 13 Zambian children will not reach their 5^th^ birthday. Studies conducted globally among displaced populations have shown that in long established refugee camps, the U5MRs are systematically lower for refugees than for surrounding host populations. In discussing disparities that exist within rural settings and displaced population camps, which are mostly set up in rural areas, provided a basis for discussing universal access to health care programmes that may be adopted in natural rural settings to address U5MR.

Zambia has hosted refugees since the early 1950’s when liberation struggle populations from her neighbours were arriving seeking political refuge. A large number of refugees in the country have come from countries including Democratic Republic of Congo, Angola, Zimbabwe, Mozambique, Rwanda and South Africa. The majority of refugees have been integrated into the local community while some populations are hosted in the two remaining Refugee camps in Zambia.

The study was undertaken in Meheba Refugee camp which is located approximately 75 km from Solwezi is demarcated into zones housing refugee populations from similar countries in specific zones. At the time of study, the camp hosted refugees from Democratic Republic of Congo (Congo DR), Rwanda, Angola and Burundi. Meheba is an established camp meaning that the refugees resident in this place have their whole lives centred on camp activities and the majority of the children in the area have only ever known the camp as their home. The camp exhibited similar characteristics to rural areas in Zambia as refugees have settled and have been resident for more than 25 years.

## Method

### Study objectives

The study objectives included the description of under-five mortality trends, profiling of priority causes of mortality, discussing health programming in the refugee camp as well as the need to develop a model to be adopted for implementation in Zambia’s rural communities.

### Study design

The study was designed as a cross sectional study looking at under five mortality among refugees for a 10 year retrospective period. The study was conducted at an ecological level; as such the sample is drawn from observation units (years) and not individuals. According to Rosenburg [6], owing to the fact that observation units of interest were time periods, there were ten observations selected, one for each year. These observations will therefore be a sample in time [6] giving a sample size of 10 and using the total populations of each period as population denominators. A 10 year period was selected to increase the precision in identifying patterns of change in mortality following years prior to the signing of MDGs. However owing to unavailability of the necessary data a seven (7) year period was reviewed. Data was analysed using STATA version 12 and Microsoft excel. Ordinary Least square regression analysis was used.

### Study site and study participants

Meheba Refugee Camp is the largest refugee settlement in Zambia housing over 15,000 refugees. This study focused on all under-five mortality cases reported during the period 2008–2014 through the camp based Health Information System (HIS) database as well as number of children under the age of five resident in the camp who were assigned refugee status at Meheba. Study participants were drawn from vital events records of births and deaths in a particular year. The HIS data is updated routinely by Health information officers at District level. There is a likelihood of under reporting in the dataset of deaths if they are not reported at facility or do not occur within the facility.

## Results

### Population distribution

The proportion of children aged 0–59 months in Meheba represented 13 % of the Camp population reflecting a significant age group whose well-being was necessary for review. The size of the population in this age group has seen periods of peaks and declines when compared amongst the years of review. The average number of children in this age group was 2943 children and the highest under five population in the camp was recorded in 2010, when about 3572 children were recorded in the camp.

### Under-five mortality

**Fig. 1:**
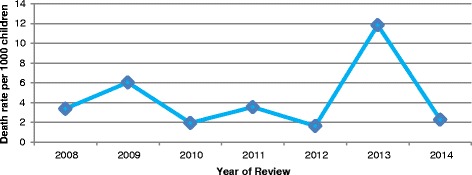
Under-five mortality (/1000 population), Meheba Refugee camp, Zambia, 2008–2014. Legend: UNHCR/Solwezi DMO HIS, 2015

The data revealed that during the period 2008–2014, a total of eighty (80) deaths were reported for children in this age group. The mortality pattern shows periods of peaks and declines over the 7 year period. The calculated average death rate was 4.4 deaths per 1000 population for the under 5 year old population. The highest rate recorded was 11.8 in 2013 and the lowest was 1.6 in 2012. Before 2013, the mortality trend showed a regressing mortality with fewer deaths being reported but changed with 2013 deaths as shown in Fig. 1.

**Fig. 2:**
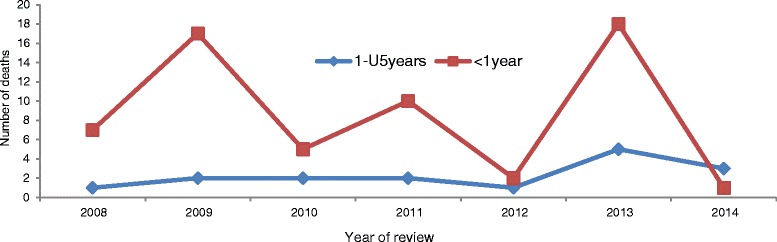
Number of deaths under the age of 5 years by age group, Meheba Refugee camp, Zambia, 2008–2014. Legend: UNHCR/Solwezi DMO HIS, 2015

To assess variability in mortality patterns, mortalities reported where disaggregated by age group as infant and child deaths respectively. The data reviewed revealed that the majority [75 %] of the deaths were reported in infant category coded as <1 year. Mortality in children aged less than 1 year (<1 year) was highest compared to that of children aged between one and less than 5 years of age whose age category showed a stable mortality pattern reported between 2009 and 2011. Figure 2 shows this mortality pattern for the less than 1 year (<1 year) and one to four (1-U5) category.

### Mortality causes

**Fig. 3:**
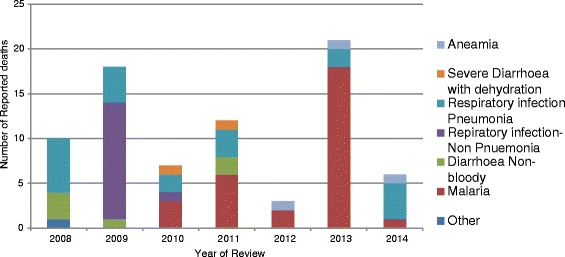
Number of Deaths by Cause under age of 5 years, Meheba Refugee camp, Zambia, 2008–2014. Legend: UNHCR/Solwezi DMO HIS, 2015

Six (6) major causes of mortality were identified and included in the analysis, namely, Pneumonia, Other respiratory infections (non-pneumonia), Malaria, Diarrhoea non bloody, severe diarrhoea with dehydration and Anaemia. An additional combined group for mortality caused by trauma, snakebites and accidents was designated as “other”. The data when analysed revealed that Malaria infection in children resulted in the 37.5 %. This is depicted in Fig. 3 which shows the mortality cause by year of review. Out of the total number of deaths caused by Malaria, about 27 % occurred in the 1–4 years age group and 73 % among infants of reported deaths between 2008 and 2014. As in the study by Moss et al. (year) on child health in complex emergencies the leading causes of mortality where Malaria [37.5 %], Pneumonia [26 %] and other respiratory infection-non pneumonia [17.5 %].

### Facility attendance

**Fig. 4:**
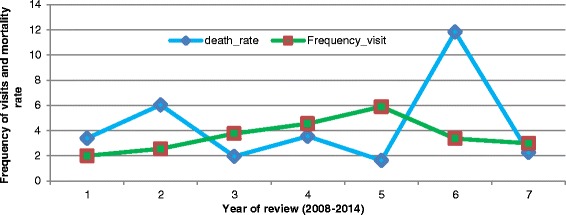
Under-five mortality rate (/1000 population) by average number of health facility attendance, Meheba Refugee camp, Zambia, 2008–2014. Legend: UNHCR/Solwezi DMO HIS, 2015

As with mortality trends, health facility attendance trends varied across the year under review showing peaks and depressed periods. The data showed an average four (4) visits per child to a facility. An attempt was made to examine differential trends between frequency of visits and the reported deaths to see if this had a bearing on number of deaths reported per year. The results of this descriptive review depicted that for years with higher mortality rates, the frequency of visits (attendance variable) was lower as compared to years with lower mortalities reported as shown in Fig. 4.

**Fig. 5:**
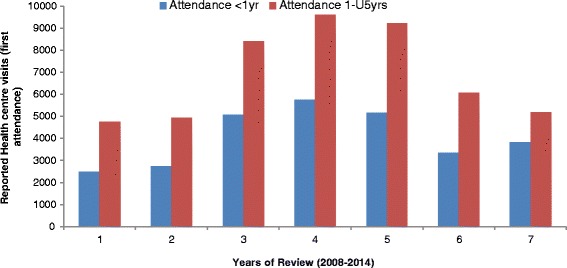
First health facility attendance by age group, Meheba Refugee camp, Zambia, 2008–2014. Legend: UNHCR/Solwezi DMO HIS, 2015

Attendance was further assessed among the age groups <1 year and the 1-U5years age group as depicted in Fig. 5. The data revealed that 63 % of all reported visits to the health facilities occurred in the 1-U5 age group. This data could suggest that the increased visits for the 1-U5 age group averted a number of deaths compared to the <1 year age group which accounted for 75 % of recorded mortalities. This variable measured as the frequency of hospital visits in a year when modelled with time and death rate showed that the reduction in mortality observed in years with higher frequency of visits was statistically significant (*P* < 0.05).

### Age at death

The descriptive review of data showed that mortality trends among the under-five age group had higher deaths reported in the <1 year group (Infant mortality) than the 1–4 years age group (child mortality).

### Ethnicity

The HIS data did not provide data on the exact country of origin for the family of the deceased child. The location of health facility and the catchment area of the facility was identified and nationality of refugees resident in an area obtained and was used as a proxy measure for ethnicity. Angolan and Congolese nationals account for the highest number of refugees in Meheba. From the data in the table most (77 %) mortalities were linked largely to Angolan nationals although exact numbers of refugees by country of origin were not available at the time of review.

Table 1 summarises key findings from the study.

**Table 1:** Mortality in under-five in Meheba Refugee camp, Zambia, 2008–2014

Variable	Category	Findings	Time/Population/deaths	Age
Ethnicity	Angolan Congolese Rwandan Burudian	77 % of deaths among Angolan refugees	2008 - 3,242 (10 deaths) 2009 – 2,983 (19 deaths) 2010 – 3,572 (7 deaths) 2011 – 3,378 (12 deaths) 2012 – 2, 439 (3 deaths) 2013 – 2,788 (23 deaths) 2014 – 2,198 (6 deaths)	<1 year- 60 deaths (75 %) reported in this age group 1-U5years- 20 deaths (25 %) reported in this age group
Attendance	Average number of visits to the health facility	Average 4 visits per child (63 % of visits occurred in 1-U5age group)		

## Discussion

The study design identified one general objective and four specific objectives in discussing correlates of under-five mortality and trends among refugees displaced in Meheba. Differential levels and trends of under-five Mortality in Meheba refugee camp were observed and assessed. The study reviewed camp registration and mortality data for children aged less than 5 years from 2008 to 2014. The camp health facilities reported a total of 80 deaths among the children in this age group. Specific years reported differences in mortality that could be attributed to various changes that the camp has continued to experience including voluntary repatriation of refugees and inflow of new refugees. Correlating mortality factors explored in the study included frequency of health facility visits, ethnicity and age.

### Age and under-five mortality

The analysis of mortality data revealed that infant mortality remains high when compared to child mortality in Meheba which is in line with ZDHS 2013–2014 findings with child mortality at 31 per 1000 births and infant mortality reported at 45 per 1000 births. Infant mortality is an important measure of the overall health status and wellbeing of the population. [7], in a study of infant mortality levels in the United Kingdom, observed that 60 % of all reported deaths in children (defined by the study as anyone aged less than 18 years) occurred before the first birthday. Infant deaths therefore reflected on the quality of midwifery, obstetric and new born care in the United Kingdom. Factors such as low birth weight, pre-term births and neonatal mortality are associated [7]. Although the Meheba study did not look at maternal health care services in the area, the high infant mortality levels could have resulted from the quality of maternal care in Meheba. Studies of health care in refugee camps have shown that 80 % of the refugee population comprises of women and children and more often maternal health infrastructure does not meet the health needs of the population [1].

### Ethnicity

The study revealed that 77 % of mortalities reported were among Angolan refugees while refugees of Rwandan origin had the lowest mortality numbers reported. According to Becher et al. ethnicity and religion show the diversity of mortality risk factors in given population. In a study of child mortality trends in Burkina Faso, it was observed that groups with low infant mortality where groups with highest proportion of Christians [2]. The Burkina Faso study found that childhood mortality risk factors in a given population were likely to be explained by several cultural differences. Owing to the fact that religion and ethnic group were correlated they were not disentangled for the study [2]. Rwanda comprises about 96.5 % of Christians which is similar to Angola. Although this study did not explore religion, it parallels the Becher study on the basis that cultural differences are likely to be mortality risk factors.

### Health service provision and attendance

Frequency of health facility visits (assessed as attendance in the study) was seen to significantly reduce the proportion of children dying in a year in the Meheba study. A study was conducted in Iran to identify risk factors of mortality among children aged 1–59 months. In this study more frequent health care visits was found to be an important protective factor in reducing mortality. The number of total child health care visits was related to under 5 year old mortality [4]. In fact, late onset of child care and/or lack of care were significant risk factors for children death [4]. Regular care visits were observed to be important in early diagnosis and treatment of children’s acquired or congenital health problems. This parallels findings for this study as frequent health facility visits where observed to reduce mortality in children under five by 3/1000.

## Conclusion

The study revealed that preventable diseases for which low cost treatment is available continue to result in under-five mortality in Mehaba refugee camp. These diseases included Malaria, Pneumonia, Diarrhoea and other respiratory infections. Findings from this study also explored interventions by the UNHCR and the MoH in reducing under-five deaths and revealed that years with higher mortality levels were met with responses around malaria control and notable increases in facility attendance by residents. Increase facility attendance was noted to reduce mortality levels to up to 3/1000 children in the camp. The study also found that infant mortality rates were higher than child mortality patterns which speak into the quality of maternal health that was not explored in this study. Displaced populations hosted in the Zambia also face similar trends in mortality patterns and more infant deaths occur compared to child mortality. Although in its post emergency phase Meheba refugee camp data revealed that mortality can be significantly reduced or controlled at minimal levels if efforts are sustained towards prevention of “killer” diseases as has been observed in the review of data in the Meheba Refugee Camp study.

## Abbreviations

CDC: Centre for disease control
CMRs: Crude mortality rates
CSO: Central statistics office
HIS: Health information system
MDGs: Millennium development goals
OPD: Out patient department
PHC: Primary health care
RHC: Rural health centre
U5MR: Under-five mortality rate
UNDP: United Nations Development Program
UNHCR: United Nations High Commission for Refugees
UNICEF: United Nations International Children’s Emergency Fund
WHO: World Health Organisation
ZDHS: Zambia demographic health survey
